# *De novo* transcriptome assembly databases for the central nervous system of the medicinal leech

**DOI:** 10.1038/sdata.2015.15

**Published:** 2015-04-28

**Authors:** Dror Hibsh, Hadas Schori, Sol Efroni, Orit Shefi

**Affiliations:** 1 Faculty of Life Sciences, Bar Ilan University, Ramat Gan 5290002, Israel; 2 Faculty of Engineering, Bar Ilan University, Ramat Gan 5290002, Israel; 3 Institute of Nanotechnologies and Advanced Materials, Bar Ilan University, Ramat Gan 5290002, Israel

**Keywords:** Transcriptomics, RNA sequencing, Central nervous system, Genome informatics

## Abstract

The study of non-model organisms stands to benefit greatly from genetic and genomic data. For a better understanding of the molecular mechanisms driving neuronal development, and to characterize the entire leech *Hirudo medicinalis* central nervous system (CNS) transcriptome we combined Trinity for *de-novo* assembly and Illumina HiSeq2000 for RNA-Seq. We present a set of 73,493 *de-novo* assembled transcripts for the leech, reconstructed from RNA collected, at a single ganglion resolution, from the CNS. This set of transcripts greatly enriches the available data for the leech. Here, we share two databases, such that each dataset allows a different type of search for candidate homologues. The first is the raw set of assembled transcripts. This set allows a sequence-based search. A comprehensive analysis of which revealed 22,604 contigs with high e-values, aligned versus the Swiss-Prot database. This analysis enabled the production of the second database, which includes correlated sequences to annotated transcript names, with the confidence of BLAST best hit.

## Background & Summary

For historic reasons, certain organisms, such as Escherichia coli, Saccharomyces cerevisiae, Arabidopsis thaliana, Caenorhabditis elegans, Drosophila melanogaster, Danio rerio or Mus musculus have gained the status of model organisms, due to their biological attributes and to technical advantages^1^. The study of these organisms has benefited from the concerted effort of a large research community allocating vast resources, resulting in a significantly larger body of genetic and genomic information concerning these organisms than any other. However, many other organisms have unique traits that make them valuable models for the study of specific biological processes. Until recently, genetic and genomic information for this latter group was scarce due to limitations of cost and labour intensity.

Next Generation Sequencing (NGS) tools have made the production of genetic and genomic information more accessible. NGS has made such research feasible for the study of all organisms, including those for whom no reference genetic data is available, the so-called non-model organisms^1^. Moreover, the innovative approaches to the analysis of these new sequencing data turned previously perceived obstacles into potentially surmountable challenges.

In the study presented here, we chose to focus on the transcriptome of the medicinal leech, *Hirudo medicinalis* CNS, which is a non-model organism, but serves as a well-studied model in neurobiology, specifically in neuronal development, regeneration and repair^2,3^. The leech CNS is composed of 6 fused ganglia at the head, 21 highly similar body ganglia and 7 fused tail ganglia^4^. Each ganglion contains approximately 200 pairs of neurons and is linked to its neighbours by thousands of axons^5^. This valuable model offers an interesting platform for use of molecular and cellular scientific methods for the evaluation of the involvement of specific cells in the regenerative processes^6^. Previous work has yielded characterization of specific genes in leech CNS^7,8^, and genes of interest were studied using the candidate gene approach^9–12^. Recently, an expression sequence tag (EST) database was constructed and is now available to the scientific community^13^. Yet, functional genomic studies in the *Hirudo medicinalis* are in their infancy^13,14^.

**Figure 1:**
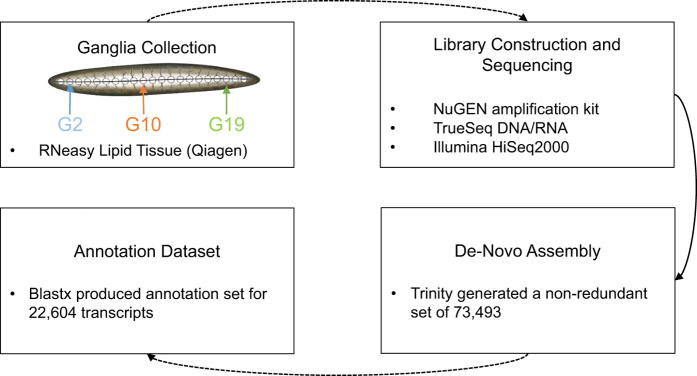
Schematic overview of the study. We collected 12 ganglia in total from the *Hirudo medicinalis*. Three biological replicates were harvested from ganglia 2, 10 and 19 and 3 technical replicates harvested from ganglion 10. Due to the low initial yield of total RNA we first amplified the RNA using NuGEN kit and then used TruSeq kit to prepare the cDNAs. Next, we sequenced the cDNAs on Illumina HISeq2000 in 50 bp single end (SE) reads. The analysis started with assembling the short reads, using the *de-novo* assembly program Trinity, and continued with functional analysis and evolutionary relationships analysis using BLASTX.

While several previous studies have shed some light on particular genes and gene expression patterns^13–15^, the full transcriptomic data of the *Hirudo medicinalis* CNS is still limited. In our related work at Bioinformatics we provided an in-depth spatial regulation analysis of the CNS transcriptome data and showed the potential of combining expression distribution patterns to produce a spatio-transcripto map along the ganglia chain^15^. As illustrated in Fig. 1, we collected RNA from three distinct locations along the leech CNS (ganglia 2, 10 and 19). To achieve the single ganglion resolution, we collected the RNA content of these organs and had to amplify the RNAs, using the NuGEN amplification kit which known to be beneficial in low amounts of RNA^16^, before sequencing. In total, we sequenced 221.1 million high-quality short reads 50 bp single-end from the leech CNS. Then, using the *de-novo* assembly program, Trinity, we reconstructed these reads to produce the first model of the leech CNS transcriptome. By combining those three distinct points along the leech CNS we assumed that our data reveals most of the transcripts that are expressed in steady state of neuronal cells in the leech CNS.

In this Data Descriptor, we provide the full assembly and annotation datasets, aimed at making our data accessible to others for use in their research and for expanding the community understanding of this data. This study complements previous approaches to address similar questions in the leech CNS^9,10,17^. Sequencing the transcriptome is a prerequisite to the expansion of our knowledge on the nervous systems (physiological and pathological conditions). Utilizing these assembly data through an annotation dataset for these new transcripts may help in the accessibility and understanding of this data. The use of a simple model, the leech CNS, together with a novel assembly and analysis approaches, combine the transcriptome with a spatial configuration, thus producing a novel transcript database of the leech CNS. Furthermore, these leech databases can be used to define the underlying conserved genetic modules controlling the equivalent patterning processes along the CNS as well as serving to cross-validate each other. Similarity, these data will offer insights into the molecular mechanism that underpin the fundamental patterning differences between leech and related organisms.

## Methods

These methods have been presented in an abbreviated form in the journal Bioinformatics^15^.

### Animal conditions

The experiments were performed on the *Hirudo medicinalis* leech. All leeches were obtained from an adult *Hirudo medicinalis* colony grown in France at Ricarimpex Farm. Further to the transportation from the farm, leeches were maintained in our animal facility in tanks populated with about 20 leeches in a controlled environment, at 16 °C and 12 h/12 h day/night cycle.

### Experimental design

Twelve samples with a focus on the *Hirudo medicinalis* CNS were taken from six different leeches for this experiment. Before use, leeches were placed on ice for 30 min and then dissected dorsally. Three ganglia (2, 10 and 19) were harvested from three leeches (Fig. 1). For technical replicas, ganglion number 10 was harvested from three additional leeches, pooled together for RNA isolation and separated into three samples for RNA-seq.

### CNS/Ganglia collection

**Figure 2:**
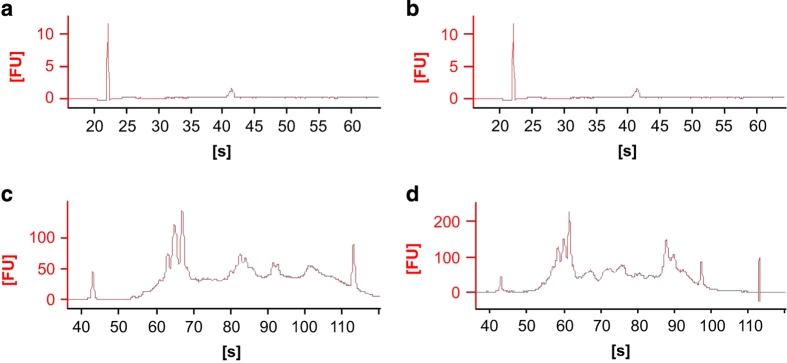
Bioanalyzer pico chip and DNA 1000 Chip analysis of total RNA output. Representative electropherogram of Bioanalyzer analysis of total RNA integrity from the 2 samples. In (**a**) and (**b**) are the pico chip analysis for 2 representative RNA samples (ganglion 10). The x axis outlines the time in seconds and the y axis provides the fluorescence. The first peak, in the ~22 s represents the ladder. The second peak in ~25 s represents the RNA. The third peak in the ~41 s represents the ribosomal RNA. For our study we selected only RNA samples that were found to have RNA concentration >45 pg/μl. In (**c**) and (**d**) are the DNA 1000 chip for 2 representative amplified cDNAs samples (ganglia 2 and 10, respectively). The first peak, in ~42 is the ladder and from ~60–110 is the cDNA. The distribution of the diagrams is due to the different sizes of the cDNAs after the amplification. All cDNAs showed concentration >72 ng/ul.

Total RNA was extracted from each ganglion using RNeasy Lipid Tissue (Qiagen). The quality and quantity of each RNA sample was assessed by Agilent’s 100 Bioanalyzer pico chip (Fig. 2a,b).

### RNA amplification

The initial RNA yield was low, requiring amplification of RNAs using a specific kit prior to the use of the mRNA-TruSeq preparation kit. RNA was amplified using Ovation Kit v2.0 (NuGEN) (Fig. 2c,d). Before amplification, all samples were lyophilized using a SpeedVac instrument and then suspended in 5 μl of nuclease-free water. This was the starting volume of NuGEN kit. Then, (as suggested by NuGEN), the 2 μg (in 100 μl) of cDNAs were fragmented by a Bioruptor instrument 3 cycles of 10 s of sonication and 90 s of pause. Library preparation proceeded with an ‘END-REPAIR’ reaction of NEB kit, then with TruSeq DNA/RNA libraries preparation according to Illumuna protocol. The 12 stock libraries were loaded on a High Sensitivity Chip and quantified on a QuBIT instrument, in order to prepare the two 6-plex pools were separated into two pools. The two pools were quantified (molarity) on Bioanalyzer HighSensitivity as stocks. In order to better balance the single libraries inside the pools, we decide to quantify libraries with qPCR, following the NuGEN suggestion for pool preparation.

### Illumina sequencing

The cDNA libraries were generated using messenger RNA-seq (mRNA-seq) assay for transcriptome sequencing on Illumina Hiseq2000 (Data Citation 1 and Table 1). Three cDNA libraries were generated from the total RNA of ganglion number 19 and three cDNA libraries were generated from the pooled total RNA of ganglion 10 in equal amounts, and sequencing was performed in one lane to generate 50 bp single end (SE) reads. A similar procedure was carried out for ganglia numbers 2 and 10. Library construction and sequencing was performed by a commercial service provider (IGA, Applied Genomics Institute).

### *De novo* assembly

Due to the fact that the genome/transcriptome of the *Hirudo medicinalis* is not available yet, we used *de-novo* tools for the reconstruction the transcriptome. The tools we used are Trinity^18^ (version trinityrnaseq_r2012-03-17) and Trans-ABySS^19^ (version 1.3.2). Here we report only the procedure and the results from Trinity, the full process and considerations can be seen in our analysis paper^15^. Trinity has been developed for assembly of short reads using de Bruijn graph algorithm by single k-mer. Trinity was executed in the inchworm method and the minimum contig length set to 200 nucleotides. The other parameters we used are default for Trinity single-end assembly (Trinity.pl—seqType fq—kmer_method inchworm—single seq.input—output seq.output—min_contig_length 200) (Data Citation 2 and Table 2).

### Annotation dataset

In general, the creation of an annotation dataset is derived from a draft genome or transcriptome^20,21^. For the *Hirudo medicinalis* none of these are available. Therefore, we used BLASTX (version 2.2.23) to identify sequence conservation and to create an annotated dataset for the 73,493 contigs, generated by Trinity, against Swiss-Prot database downloaded from the National Center for Biotechnology Information (NCBI). We used blast commands setting the minimum e-value to be *e* ^−10^ for maximal confidence of the contigs (blastall -p blastx -i Trinity.fasta -d Swiss-prot *e* ^−10^ −o Trinity.fasta.out). Next, to improve the readability of blast output we kept only the first result for each contig by using Linux common command (grep -E ‘Query\=|No\ hits\ found|^gi\|’ Trinity.fasta.blastx.out|grep -A1 ‘Query\=\contig_name’| sed ‘/^--/d’| sed ‘s/path.*//’ | paste - - -d’;’Trinity.fasta.blasx.out.best-hit). Applying the described pipeline has led to a set of reliable annotated contigs (Data Citation 3).

## Data Records

In this study we deposited four datasets. The first dataset is the RNA-Seq raw reads (Data Citation 1 and Table 1). This dataset contains 12 samples in total. Three biological replicates from each ganglia (numbers 2, 10 and 19), and three more technical replicates from ganglion number 10. The second dataset is the expression value of each of the Trinity assembly contigs (Data Citation 2 and Table 2). The third dataset contains the actual contigs (Data Citation 2 and Table 3). The fourth dataset is the annotation file (Data Citation 3). The annotation file is a comma separated value (CSV) format file with all of the annotated contigs generated by Trinity. The annotation file deposited in figshare depository. In general, there are 7 columns. Column #1 is the name of the contig, #2 is the length, #3 is the gi accession number, #4 is the sp accession number, #5 is the entry name, #6 is the BLASTX score and #7 is the e-value. The first two datasets described above (Data Citation 1 and Table 1, and Data Citation 2 and Table 2) were previously published in our related work in the journal Bioinformatics^15^, and the third dataset in Data Citation 2 (Table 3) and the forth dataset in Data Citation 3 are the core of this work and have not been published before.

## Technical Validation

### CNS collection quality control

Prior to processing, after samples were harvested, we assessed the quality and quantity of each sample using Agilent’s 100 Bioanalyzer pico chip. Only RNA samples which contained a concentration of 45–170 pg/μl were used for this experiment (Fig. 2a,b). Since the harvesting was only from single ganglions (~400 cells), we expected low concentrations of total RNA.

### RNA amplification quality control

Following amplification, cDNAs were quantified using Nanodrop and Bioanalyzer DNA 1000 Chip (Fig. 2c,d). After balancing the two pools for a similar total pool volume, we proceeded with the procedure. The first pool volume was 47.2 μl and the second pool was 45.9 μl.

### Sequencing quality control

We used multiple steps for testing sequencing quality. The first step included a count of total reads and total bases for each of the samples to ensure that the amounts are approximately of the same order of magnitude. These amounts were 10–26 million reads. As a second step, we tested samples to pass FastQC^22^ for basic statistics as quality estimation, per base sequence quality, per sequence quality score, length distribution and raw reads quality control (Table 4). As a third step, we estimated the sequencing depth of coverage by estimating the size of the transcriptome. We used the know information on a related organism, *Helobdella robusta*, which its transcriptome size is estimated to be 29032248 bp (http://metazoa.ensembl.org/Helobdella_robusta/Info/Annotation/#assembly). Thus, we estimated the sequencing depth of coverage to be $\frac{11000000000\;\mathrm{bases}}{29032248}=378$ (the number of sequenced bases divided by the predicted transcriptome size).

### Assembly quality control

**Figure 3:**
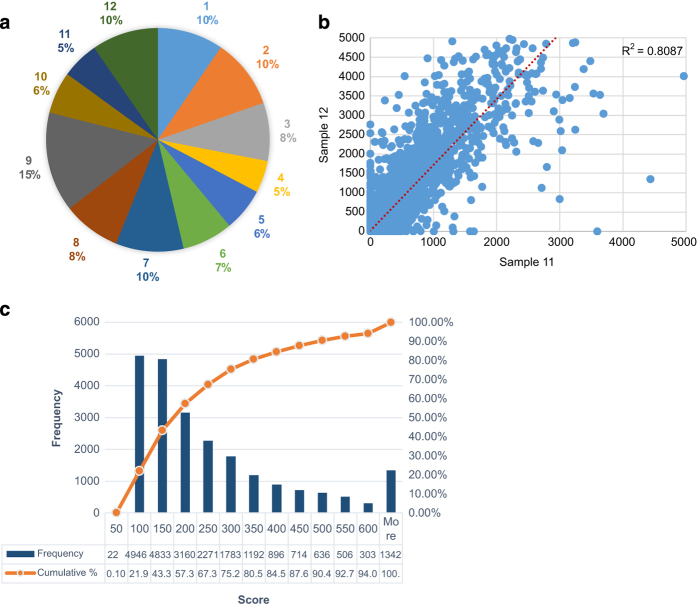
Quality control at Sample level following sequencing. In (**a**) is the samples’ contribution to the assembly. The percentage in the pie chart outlines the percentage that each sample contributed to the reconstruction of the transcriptome dataset. The numbers in the pie chart outline the sample number. Out of all the 73,493 contigs, sample number 5 contributed to reconstruct 5% and sample number 9 contributed 15%. As can be seen in the chart, the percentiles vary from 5 to 15% and the dataset is not biased towards any specific sample. In (**b**) is a scatter plot of the technical replicates expression values for ganglion number 10 to verify that the samples sequenced without bias. The x axis outlines sample number 11 expression values, which represent technical replica number 2. The y axis provides sample number 12 expression values, which represent technical replica number 3. In the top right is the *R*^2^ correlation. We also tested the correlation between technical replica 1 to 2 and 1 to 3, and they have also showed strong *R*^2^, 0.789 and 0.7845 respectively. In (**c**) is the hits Scores Distribution. The x axis outlines the upper limit of the score. The left y axis provides the frequency, and the right y axis provides the cumulative. Out of the 73,493 transcripts reconstructed by Trinity 22,604 had high e-value hits in the Swiss-Prot database. The blast score gives an indication of the quality of the alignment. As can be seen, most transcripts aligned with a score higher than 50 and only 22 had a score under/equal 50.

Following the use of Trinity, to make sure that the produced contigs are correct, we compared our model of transcriptome also to *Helobdella robusta* published transcriptome. We start with comparing basic statistics such as average length of contigs (Table 4). We calculated the average contig length for the *Hirudo medicinalis* and we found it to be 1124 (Table 5), which is very similar to the average transcript size for the *Helobdella robusta* which is 1239 according to the Joint Genome Institute (http://genome.jgi-psf.org/Helro1/Helro1.home.html). Next, we used Bowtie^23^ to map reads back to the contigs to test the mapping rate for each sample (Fig. 3a). Finally, we correlated the technical replicates between themselves, which showed a strong correlation (Fig. 3b) (Table 3). Moreover, for examining the completeness of the data we tested full-length over the innexin genes family to see if the transcripts are really full without any bias to particular side, and we found that 11 out of the 21 innexin reconstructed perfectly, and the other 10 almost in fully^15^. Moreover, for strengthening the completeness evaluation of our data we estimated the transcriptome size according to the EnsemblMetazoa known information of the *Helobdella robusta* (see above), and found that the number of coding genes for the *Helobdella robusta* is 23432 which is very similar to the number of contigs with high e-value that when aligning to the Swiss-Prot (Fig. 3c).

### Annotation quality control

To ensure the quality of our annotation we set the blast e-value threshold to *e* ^−10^. We found that the blast scores were high (Fig. 3c). By counting the number of transcripts that passed the threshold, we saw that the approximate predicted size of the *Hirudo medicinalis* CNS transcriptome is 22,604. This result is very similar to size of the *Helobdella robusta* and the *Capitella teleta* transcriptomes (19,487 and 37,908, respectively).

## Usage Notes

The data provided in this experimental set can be used for several purposes. First, it is possible to use the raw reads for executing a new experiment, with different analysis approaches. Second, each analysis step can be performed differently as all the technical experimental information is publicly available.

### *De novo* assembly

By using our non-redundant set of 73,493 transcripts we generated using Trinity, the search in the dataset for genes of interest can be easily done by homologs using blast, or text based search if using the annotation table. Moreover, the raw reads provided here make it possible to use alternative methods for *de novo* assembly that may assemble the reads into a different set of transcripts than the ones we constructed. The *de novo* assembly can be performed using SOAPdenovo-Trans^24^ or Velvet/Oases^25,26^.

### Differential expression

The combination of raw reads and transcripts allows the re-calculation of expression values for each transcript for each sample. There are different approaches for discovering differentially expressed genes. This can be further refined into new groups of genes expressed in each individual ganglion, or combinations of ganglia. This can be performed by using, for example, free Bioconductor packages such as DESeq^27^ or edgeR^28^ (http://www.bioconductor.org/).

### Downstream analysis

The datasets provided here represented by 3 biological replicates for each condition and technical replicates for one condition. Comparing the ganglia sequenced here to other ganglia to determine the set of genes statistically significantly differentially expressed along the whole CNS, and not only three points.

## Additional information

**How to cite this article:** Hibsh, D. *et al.* *De novo* transcriptome assembly databases for the central nervous system of the medicinal leech. *Sci. Data* 2:150015 doi: 10.1038/sdata.2015.15 (2015).

## Supplementary Material



## Figures and Tables

**Table 1:** Raw data deposit

**Sample no.**	**GEO Sample**	**SRA Runs**	**BioSample**	**Title**
1	GSM1109855	SRR799260	SAMN01993646	1_ganglion-number_2
2	GSM1109856	SRR799261	SAMN01993647	2_ganglion-number_10
3	GSM1109857	SRR799262	SAMN01993648	3_ganglion-number_19
4	GSM1109858	SRR799263	SAMN01993649	4_ganglion-number_2
5	GSM1109859	SRR799264	SAMN01993650	5_ganglion-number_10
6	GSM1109860	SRR799265	SAMN01993651	6_ganglion-number_19
7	GSM1109861	SRR799266	SAMN01993652	7_ganglion-number_2
8	GSM1109862	SRR799267	SAMN01993653	8_ganglion-number_10
9	GSM1109863	SRR799268	SAMN01993654	9_ganglion-number_19
10	GSM1109864	SRR799269	SAMN01993655	10_ganglion-number_10
11	GSM1109865	SRR799270	SAMN01993656	11_ganglion-number_10
12	GSM1109866	SRR799271	SAMN01993657	12_ganglion-number_10
This dataset contains 12 samples in total. Three biological replicates from each ganglia (numbers 2, 10 and 19), and three more technical replicates from ganglion number 10. Samples 1–3 are from animal 1, samples 3–6 are from animal 2 and samples 7–9 are from animal 3. Samples 10–12 are from 3 additional different animals. The sequenced data were deposited at Gene Expression Omnibus (record GSE45569 or in Sequence Read Archive (SRA) accession number SRR799260-71) (Data Citation 1).				

**Table 2:** Expression data deposit

**GEO Series**	**File name**	**File type**	**Data**
GSE45569	GSE45569_counts_processed_data_files	Supplementary	Expression
Each column in this file contains the expression value, calculated by RSEM, of a specific contig across the 12 samples. The ID in this file is the name of a contig given by the *de novo* assembly of Trinity (Data Citation 2).			

**Table 3:** Contigs deposit

**SUBID**	**BioProject**	**BioSample**	**Accession**
SUB652952	PRJNA195129	SAMN03003628	GBRF00000000
The dataset contains the contigs generated by Trinity, Transcriptome Shotgun Assembly project, has been deposited at DDBJ/EMBL/GenBank under the accession GBRF00000000. The version described in this paper is the first version, GBRF01000000 (Data Citation 2).			

**Table 4:** Raw reads quality control

**Sample no.**	**Q20**	**Q30**	**Minimum**	**Maximum**	**Average**
1	0.980327578	0.857704845	2	39	34.32561514
2	0.99457281	0.969370342	2	39	37.60889307
3	0.993736658	0.967448115	2	39	37.56479201
4	0.991925529	0.969219125	2	39	37.76659668
5	0.989656355	0.906750074	2	39	35.28654572
6	0.991760698	0.96556944	2	39	37.62150186
7	0.981892325	0.964431624	2	40	37.56354729
8	0.988048156	0.960159186	2	39	37.53777952
9	0.987755796	0.892754278	2	39	34.97012768
10	0.993808588	0.928533237	2	39	35.70516752
11	0.988451201	0.898891407	2	39	35.11481731
12	0.987864211	0.972036737	2	40	37.72294809
The sample numbers are the same as described in Table 1. The Q20 and Q30 calculated from the FastQC output for each sample separately. The minimum, maximum and average are phred score quality indicators.					

**Table 5:** Assembly statistics

**Category**	**Trinity**
Number of contigs	76845
Average contig length	1124
N25	4352
N50	2188
N75	916
s.d.	1615
Total length of transcriptome	86436165
The statistics presented here are for Trinity contigs prior to filtering out low quality contigs according to the transcriptome shotgun assembly (TSA) criteria.	
